# Inflammatory Myofibroblastic Tumor of the Temporal Bone: A Case Report

**DOI:** 10.7759/cureus.117513

**Published:** 2026-10-05

**Authors:** Morio Yamaguchi, Hirotaka Ikeda, Tsukasa Kuno, Hayato Takahashi, Atsuhiro Tsuda, Suguru Miyachi, Yuya Nishiyama, Satoshi Yoshioka, Seiji Yamada, Masanori Inoue

**Affiliations:** 1 Department of Radiology, Fujita Health University School of Medicine, Toyoake, JPN; 2 Department of Neurosurgery, Fujita Health University, Toyoake, JPN; 3 Department of Otolaryngology - Head and Neck Surgery, Fujita Health University School of Medicine, Toyoake, JPN; 4 Oncology Innovation Center, Research Promotion Headquarters, Fujita Health University, Toyoake, JPN

**Keywords:** computed tomography, imaging-histopathologic correlation, inflammatory myofibroblastic tumor, magnetic resonance imaging, temporal bone

## Abstract

Inflammatory myofibroblastic tumor (IMT) is classified as an uncommon intermediate-grade neoplasm that rarely involves the temporal bone. We report a case of a 23-year-old female who presented with right aural fullness and pain. Computed tomography findings showed a 1.6-cm well-circumscribed soft tissue mass in the superior mastoid portion of the right temporal bone, with bone destruction extending to the tegmen mastoideum. Magnetic resonance imaging (MRI) showed T1 isointensity and predominantly mild T2 hyperintensity with internal hypointense areas. Two months later, preoperative MRI showed interval enlargement of the mass to 2.4 cm, with heterogeneous enhancement and adjacent dural thickening and enhancement. The biopsy results showed proliferation of the spindle cells with inflammatory cell infiltration, and the tumor cells were positive for anaplastic lymphoma kinase (ALK), supporting the diagnosis of IMT. Histopathologic evaluation of the resection specimen revealed collagen-rich fibrous components and myxoid stroma, mixed inflammatory infiltrates, hemorrhage, hemosiderin deposition, and focal dural invasion without brain involvement. The patient remained recurrence-free five years after surgery. The heterogeneous T2 signal may reflect the underlying histologic heterogeneity of the tumor. IMT may be considered in young patients with a bone-destructive temporal bone mass showing heterogeneous T2 signal, contrast enhancement, and interval enlargement. Furthermore, histopathologic evaluation is essential to reach a definitive diagnosis.

## Introduction

Inflammatory myofibroblastic tumor (IMT) is a rare soft tissue neoplasm that is characterized by the proliferation of myofibroblastic and fibroblastic spindle cells accompanied by an inflammatory infiltrate predominantly comprising plasma cells, lymphocytes, and eosinophils [1]. It most commonly occurs in children and young adults, with a predilection for the abdominal cavity, retroperitoneum, and lungs. Involvement of the head and neck is relatively uncommon [2-5], and temporal bone involvement is particularly rare [6].

IMT is classified as an intermediate-grade neoplasm: distant metastasis is rare, but local recurrence and invasive growth can occur [1]. A subset of IMTs harbor rearrangements involving the anaplastic lymphoma kinase (ALK) gene, and ALK immunohistochemistry can provide important diagnostic support [4]. Historically, the term inflammatory pseudotumor (IPT) has been used for a heterogeneous group of lesions that included IMT, although these terms are not considered synonymous in current classification [4].

The imaging findings of the temporal bone IMT are variable, including bone destruction, diverse magnetic resonance imaging (MRI) signal characteristics, and variable enhancement. Thus, a diagnosis based on imaging alone is difficult [6-8]. This study aimed to report a case of temporal bone IMT that showed substantial interval enlargement and heterogeneous signal intensity on T2-weighted images, and to review the literature with particular emphasis on imaging-histopathologic correlations and comparison with previously reported cases.

## Case presentation

A 23-year-old female developed a sensation of fullness in the right ear. Approximately one month later, she developed a headache and right otalgia and visited a local clinic, where an MRI revealed a mass in the right temporal bone. Later that month, a CT scan performed at another clinic demonstrated associated bone destruction. Approximately one month after the onset of headache and otalgia, she was referred to the Department of Otolaryngology - Head and Neck Surgery at our hospital for further evaluation. She presented with headache, right otalgia, and aural fullness. No dizziness or vertigo was reported. Otoscopic examination showed clear tympanic membranes bilaterally. Pure-tone audiometry showed hearing within normal limits bilaterally, with pure-tone averages of 10.0 dB HL in the right ear and 11.7 dB HL in the left ear. The patient’s medical and family histories were unremarkable, and she was otherwise healthy. Laboratory tests at presentation showed no significant abnormalities, including no evidence of systemic inflammation. No neurological symptoms were documented.

Non-contrast neck CT and MRI were performed for further imaging evaluation. CT findings revealed a 1.6 × 1.3 × 1.4 cm mass with associated bone destruction in the superior aspect of the mastoid portion of the right temporal bone. The mass showed soft tissue attenuation without calcification (Figure 1A). The margins of the bone destruction were smooth (Figure 1B), with no periosteal reaction. The surrounding bone appeared normal, without abnormal sclerosis or ground-glass appearance. The bone destruction extended to the tegmen mastoideum, whereas the ossicles, tympanic cavity, and inner ear structures were preserved (Figures 1C, 1D).

**Figure 1:**
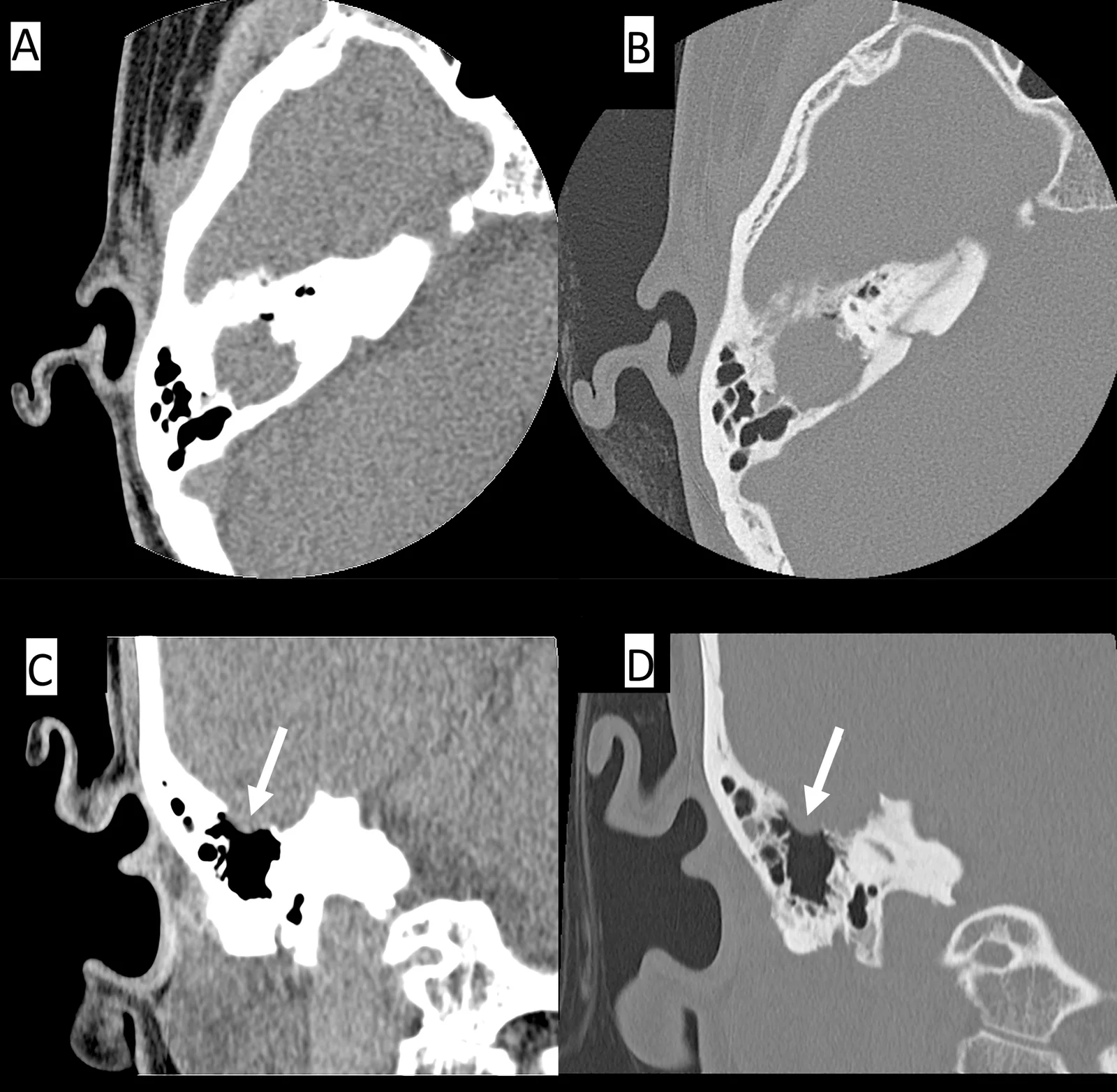
CT images: (A) axial soft tissue window image, (B) axial bone window image, (C) coronal soft tissue window image, and (D) coronal bone window image. A soft tissue attenuation mass with bone destruction is observed in the superior aspect of the mastoid portion of the right temporal bone. The margins of the bone destruction are smooth, and the surrounding bone is normal. The bone destruction extends to the tegmen mastoideum (arrow), whereas the ossicles, tympanic cavity, and inner ear are preserved.

Based on the MRI findings, the lesion appeared as a well-circumscribed, round mass abutting the dura superiorly. It was predominantly mildly hyperintense on T2-weighted images, with internal hypointense areas (Figure 2), and homogeneously isointense to the brain parenchyma on T1-weighted images.

**Figure 2:**
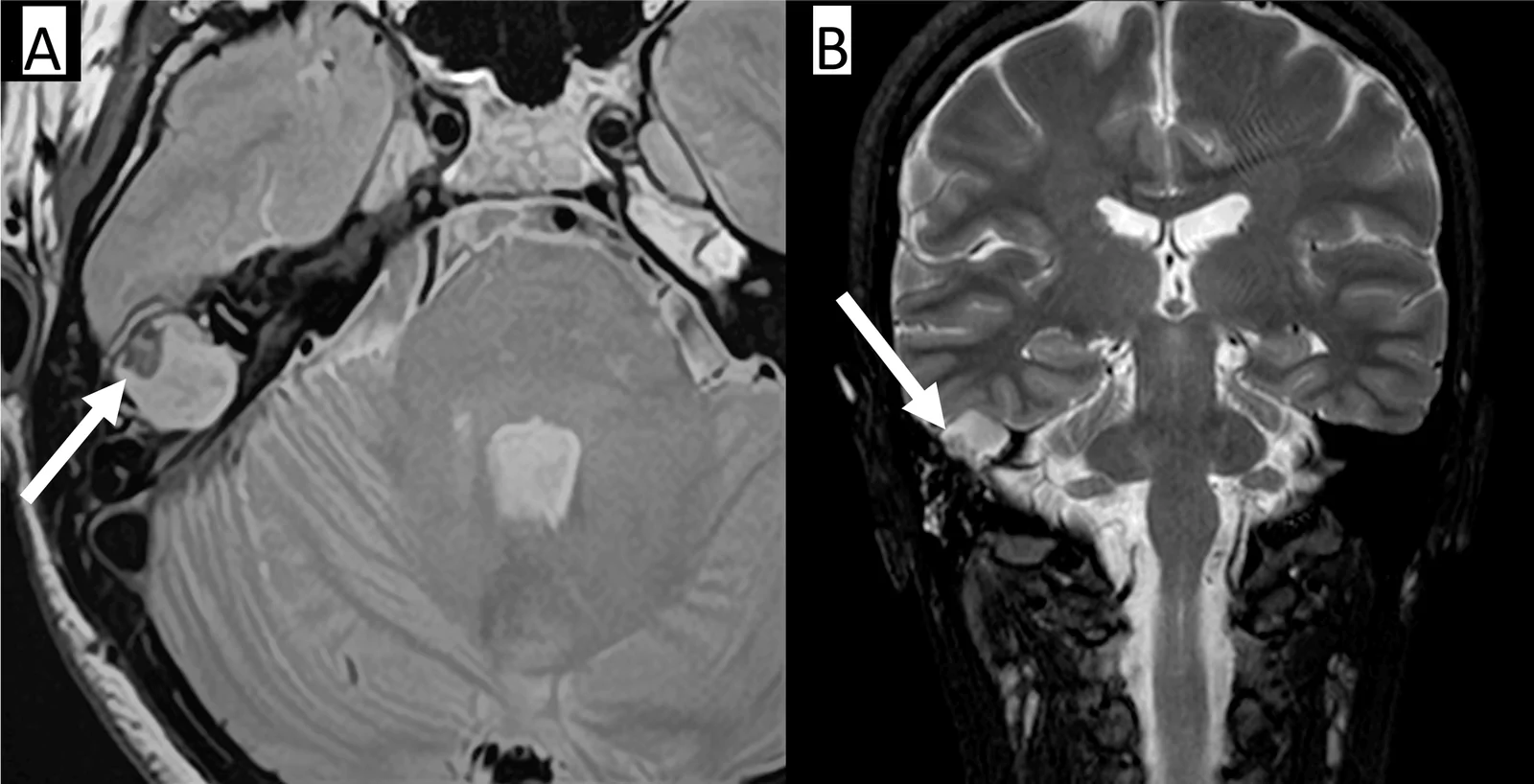
T2-weighted images: (A) axial 3D T2-weighted image and (B) coronal T2-weighted image. A 1.6 × 1.3 × 1.4 cm mildly hyperintense mass is observed in the right temporal bone, with internal hypointense areas (arrow).

Moreover, the lesion was hyperintense on diffusion-weighted images, with a minimum apparent diffusion coefficient of 0.905 × 10^-3^ mm^2^/s. The differential diagnosis included Langerhans cell histiocytosis, rhabdomyosarcoma, and cholesterol granuloma. Since the imaging findings were not diagnostic, a burr-hole biopsy was performed approximately one month after the initial evaluation at our hospital.

Histopathologic examination of the biopsy specimen showed a proliferation of spindle cells in a collagenous to myxoid stroma accompanied by an inflammatory infiltrate composed mainly of lymphocytes and plasma cells, with scattered eosinophils (Figure 3A). The spindle cells showed only mild cytologic atypia and were immunoreactive for ALK (clone D5F3) (Figure 3B), whereas CD1a, S100 protein, MyoD1, and myogenin were negative. These morphologic and immunohistochemical findings were consistent with IMT.

**Figure 3:**
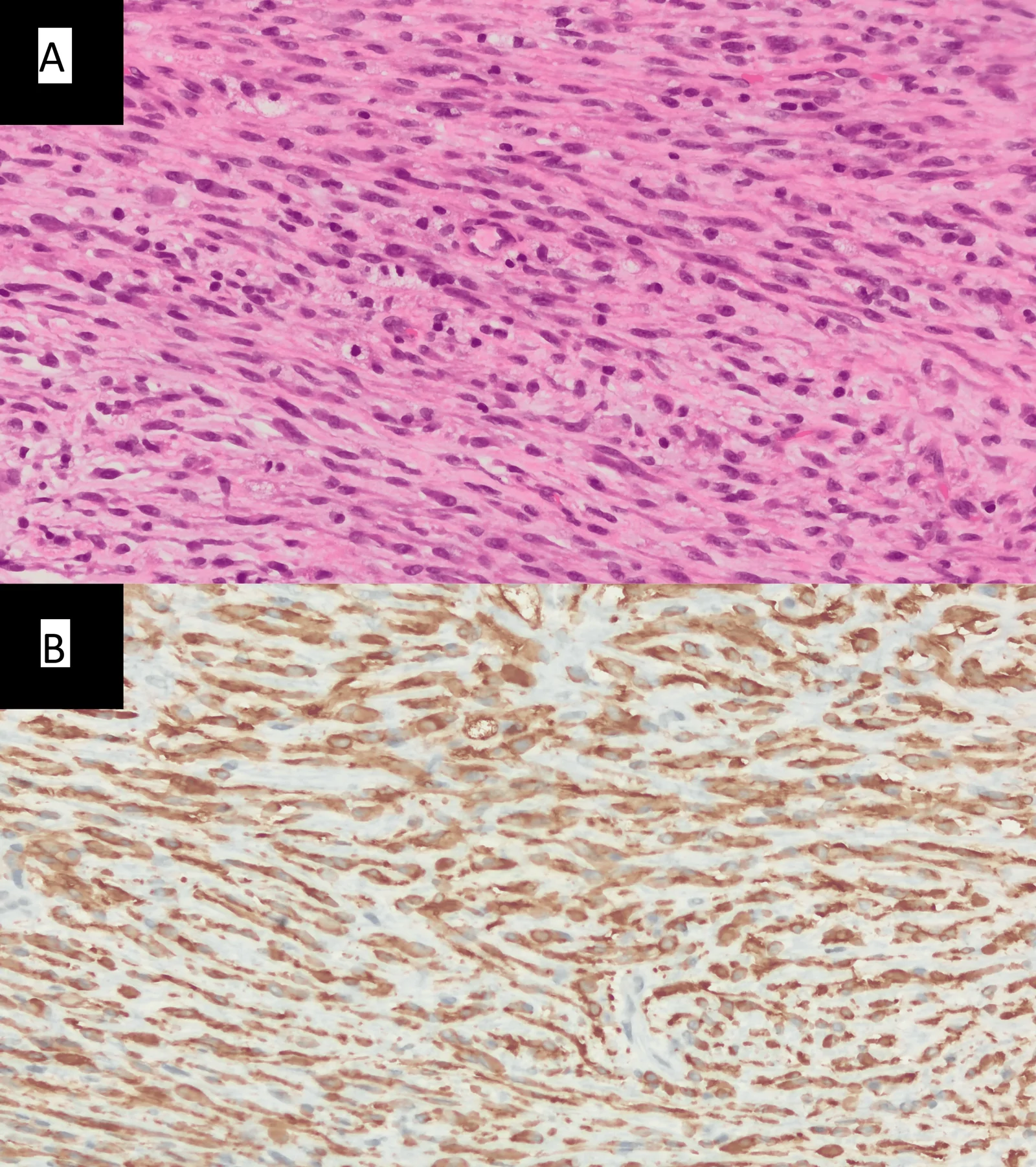
Histopathologic findings of the biopsy specimen. (A) Hematoxylin and eosin staining (original magnification, ×200). Spindle-cell proliferation in a collagenous stroma is accompanied by an inflammatory infiltrate predominantly comprising lymphocytes. (B) Anaplastic lymphoma kinase (ALK) immunohistochemical staining (original magnification, ×200). The spindle cells show cytoplasmic positivity for ALK (clone D5F3).

Approximately one month after the biopsy and two months after the initial MRI at our hospital, contrast-enhanced MRI was performed for preoperative evaluation. The mass had enlarged to 2.4 × 2.3 × 2.3 cm and showed heterogeneous enhancement (Figure 4).

**Figure 4:**
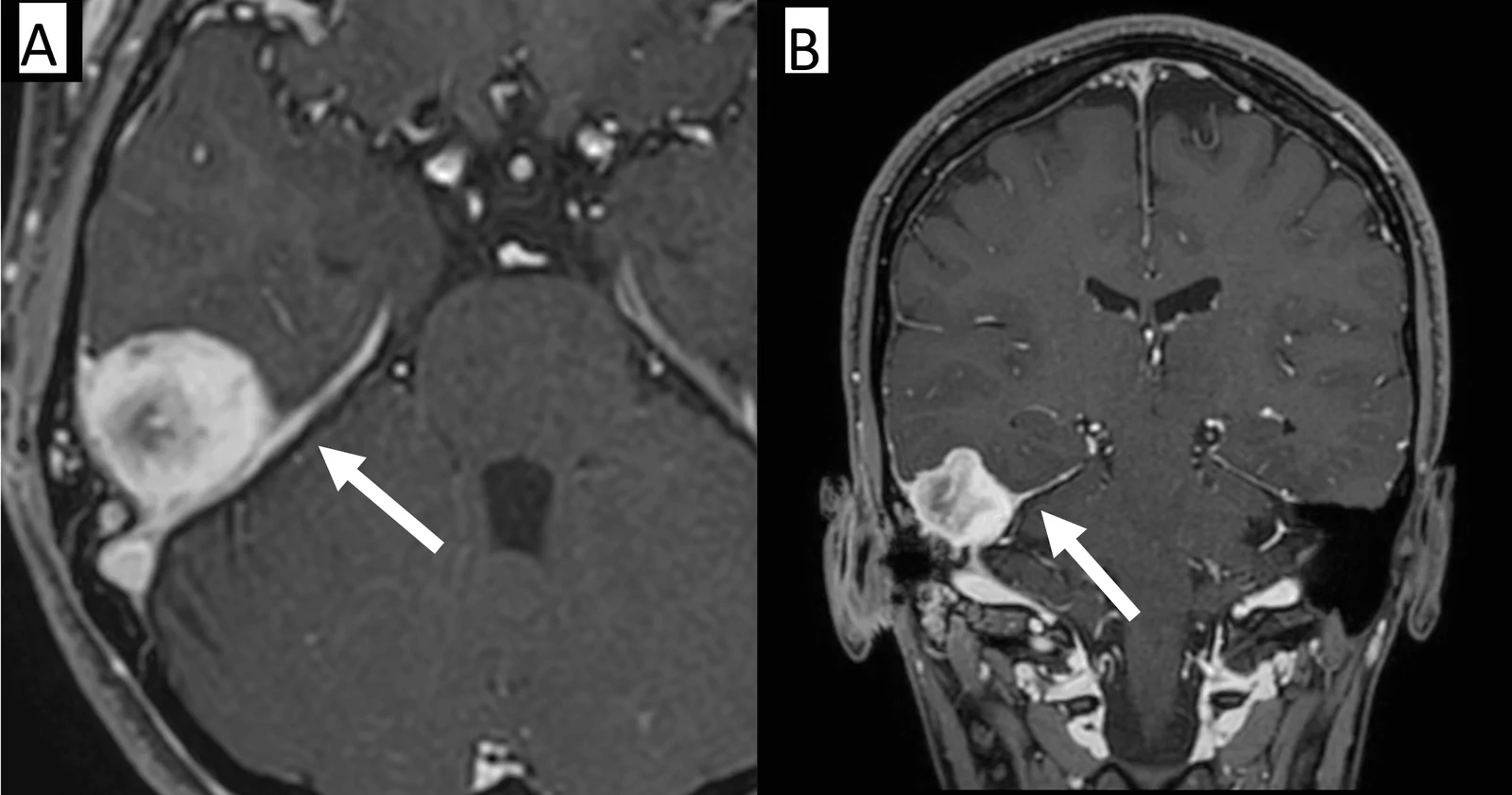
Contrast-enhanced T1-weighted images obtained two months later: (A) axial image and (B) coronal image. The mass has enlarged to 2.4 × 2.3 × 2.3 cm and shows heterogeneous enhancement. The adjacent dura is mildly thickened (arrow) and enhanced.

The adjacent dura was mildly thickened and enhanced. No abnormal enhancement was observed in the brain parenchyma, with no evidence of parenchymal invasion.

Gross total resection was performed jointly by the otorhinolaryngology and neurosurgery teams. Although the tumor was not adherent to the brain parenchyma, it invaded the dura and extended into the subdural space. Therefore, the tumor was removed together with the involved dura. The histopathologic examination of the dura adjacent to the lesion revealed tumor cell infiltration into the dura.

The subsequently resected specimen showed essentially the same histologic and immunohistochemical features, with heterogeneous collagen-rich fibrous and myxoid areas, as well as foci of hemorrhage and hemosiderin deposition. No mitotic figures or tumor necrosis were identified. Focal infiltration into the adjacent dura was present. The surgical margins on the cerebral and dural aspects were negative. However, the margin on the petrous bone side could not be reliably assessed because the tumor was exposed at the specimen surface. The patient remained asymptomatic during postoperative follow-up, and annual contrast-enhanced MRI showed no evidence of recurrence for five years.

## Discussion

Few reports have described the imaging findings of temporal bone IMT in detail. Considering the limited literature on temporal bone IMT and the historical overlap in terminology with IPT, previous reports on temporal bone IPT were also considered. These reports suggest that such lesions may appear on CT as soft tissue masses occupying the middle ear cavity and mastoid air cells, with sclerosis, erosion, or destructive changes in the surrounding bone [6,7,9,10]. The extent of osseous change varies among cases, ranging from relatively well-defined localized lesions to infiltrative lesions extending into the petrous temporal bone, internal auditory canal, dura, intracranial compartment, and adjacent skull base spaces [7,9,10]. On MRI, these lesions typically manifest as isointense or hypointense on T1-weighted images [6,8-10]. In contrast, signal intensity on T2-weighted images is variable. Although marked T2 hypointensity has been reported relatively frequently [9,10], some lesions show iso- to hyperintensity [7,10]. T2 hypointensity is thought to reflect fibrosis and collagen deposition within the lesion [6,9]. Enhancement is common on contrast-enhanced MRI, although its uniformity and degree are variable [8-10]. Thickening and enhancement of the adjacent dura may also be observed, potentially complicating differentiation from malignant tumors and infectious lesions [6,9].

In the present case, the CT findings revealed a relatively well-defined lesion with temporal bone destruction. Based on the MRI findings, the lesion was isointense on T1-weighted images and predominantly mildly hyperintense on T2-weighted images, with heterogeneous contrast enhancement. These findings were within the spectrum of imaging appearances previously reported for temporal bone IMT and related IPT. The lesion also showed substantial interval enlargement over approximately two months. However, because the follow-up MRI was obtained after biopsy, the observed increase in size cannot be attributed solely to tumor growth. Local invasion and interval growth have also been reported in temporal bone IMT [7]. Despite the interval enlargement and dural invasion, marked cytologic atypia, mitotic figures, and tumor necrosis were not identified histologically.

The histologic features of IMT are generally classified into three basic patterns: a myxoid/vascular pattern, a compact spindle-cell pattern, and a hypocellular fibrous pattern. These patterns often coexist within the same tumor [3,4,11]. In the present case, the lesion was predominantly mildly hyperintense on T2-weighted images, with intermixed hypointense areas. The resection specimen showed proliferation of the spindle cells in a background of collagen fibers and myxoid stroma, accompanied by a mixed inflammatory infiltrate comprising eosinophils, lymphocytes, and plasma cells. Previous studies have suggested that lesions with abundant myxoid-vascular components tend to show relative hyperintensity on T2-weighted images, whereas those with abundant spindle-cell or fibrous components tend to show hypointensity [6,12]. Accordingly, the mildly hyperintense signal on T2-weighted images in this case may have reflected myxoid and inflammatory components, whereas the internal hypointense areas may have reflected collagen-rich fibrous components. Hemosiderin deposition was also observed in the resection specimen and may have contributed to the hypointense areas. Thus, the heterogeneous signal intensity on T2-weighted images may reflect the histologic heterogeneity of the tumor. However, since a direct spatial correlation between the MRI signal-intensity areas and histopathologic findings was not performed, this interpretation remains inferential.

In a bone-destructive soft tissue lesion of the temporal bone, such as that observed in the present case, a definitive diagnosis of IMT based on imaging alone is difficult to reach. In young patients, the differential diagnosis may include Langerhans cell histiocytosis, rhabdomyosarcoma, cholesterol granuloma, and other inflammatory or neoplastic lesions, depending on lesion location and MRI characteristics. In the present case, the combination of heterogeneous T2 signal intensity, enhancement, adjacent dural thickening, and osseous destruction was not specific for IMT. Histopathologic evaluation, including ALK immunohistochemistry in the appropriate morphologic context, was therefore essential for diagnosis.

## Conclusions

IMT of the temporal bone is rare, and its imaging findings are nonspecific. In the present case, the lesion showed heterogeneous signal intensity on T2-weighted images, with hypointense areas, while the result of the histopathologic examination indicated histologic heterogeneity, including myxoid stroma and collagen-rich fibrous components. These findings suggest that the heterogeneous T2 signal may reflect the histologic heterogeneity of the tumor. The lesion also showed substantial interval enlargement and dural involvement. In young patients with a bone-destructive soft tissue lesion of the temporal bone, IMT may be considered in the differential diagnosis, and histopathologic evaluation should be pursued to reach a definitive diagnosis.
